# Translocation (6;15)(q12;q15): A Novel Mutation in a Patient with Therapy-Related Myelodysplastic Syndrome

**DOI:** 10.1155/2015/318545

**Published:** 2015-12-21

**Authors:** Saba F. Ali, Rebecca J. Sonu, Denis M. Dwyre, Brian A. Jonas, Hooman H. Rashidi

**Affiliations:** ^1^Department of Pathology and Laboratory Medicine, University of California Davis Medical Center, Sacramento, CA 95817, USA; ^2^Department of Internal Medicine, Division of Hematology and Oncology, University of California Davis Medical Center, Sacramento, CA 95817, USA

## Abstract

Most myelodysplastic syndromes (MDS) present with loss or gain of chromosomal material and less commonly show translocations as a sole abnormality. In addition, certain translocations are more commonly seen in MDS than others, but to our knowledge, the presence of t(6;15) has not been reported in MDS, specifically therapy-related MDS (t-MDS) cases. Patients with t-MDS, a group of heterogeneous stem cell related disorders resulting as a latent complication of cytotoxic and/or radiation therapy, generally tend to have a poorer prognosis than* de novo* MDS. We present a unique case of a patient who initially presented with acute myeloid leukemia (AML) with a normal karyotype and FLT3-ITD and NPM1 mutations. The patient was successfully treated with chemotherapy and an autologous bone marrow transplant but subsequently developed a new FLT3-ITD negative t-MDS with a unique translocation, t(6;15)(q12;q15), three years after transplant. To our knowledge, this unique sole translocation has never been reported in MDS or t-MDS and given her successful response to treatment and remission, presence of this translocation may have some prognostic value.

## 1. Case Presentation

**Figure 1:**
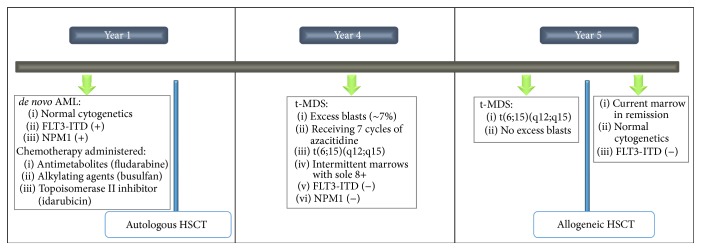
Summary of patient's clinical history. AML, acute myeloid leukemia; FLT3-ITD, Fms-related tyrosine kinase 3-internal tandem duplication; t-MDS, therapy-related myelodysplastic syndrome; NPM1, nucleolar phosphoprotein B23; HSCT, hematopoietic stem cell transplantation; t, translocation.

A fifty-one-year-old female had an initial diagnosis of acute myeloid leukemia with normal cytogenetics (46, XX) and FLT3-ITD and NPM1 mutations for which she achieved complete remission after the administration of induction chemotherapy followed by consolidation with cytarabine and an autologous hematopoietic stem cell transplantation (HSCT). Three years after her initial diagnosis, she presented with new cytopenias (leukopenia and thrombocytopenia). A subsequent bone marrow evaluation demonstrated findings consistent with myelodysplastic syndrome (MDS), and given the prior history, this new MDS was best characterized per World Health Organization (WHO) 2008 criteria as therapy-related myelodysplastic syndrome (t-MDS). The cytogenetics findings from one bone marrow showed a new cytogenetic abnormality of t(6;15)(q12;q15) with a different molecular signature compared to the patient's original acute myeloid leukemia (FLT3-ITD and NPM1 mutation negative). The unique cytogenetics and the molecular profile are consistent with a new t-MDS (unrelated to the patient's known AML). Thereafter the patient received seven cycles of azacitidine and subsequently underwent a matched unrelated donor allogeneic HSCT. With this therapy, she successfully achieved complete remission for a second time.  Figure 1 summarizes the patient's overall clinical history.

The patient's initial AML presented with flu-like symptoms, fever, and cough with a complete blood cell count (CBC) showing a background of anemia and thrombocytopenia with marked leukocytosis (249 × 10^9^/L) which were predominantly comprised of blasts (93%). She was then treated with hydroxyurea and three leukoreduction procedures via apheresis in order to prevent potential leukostasis. After the leukoreduction, a subsequent bone marrow biopsy showed a markedly hypercellular marrow (100% of total cellularity) consisting almost entirely of diffuse sheets of blasts. The blasts were intermediate in size with mildly irregular vesicular nuclei, inconspicuous nucleoli, and small to moderate amounts of cytoplasm with no identifiable Auer rods. Flow cytometry of the marrow showed that the blasts were dim CD45 positive and positive for CD117, CD13, CD33, and CD38 and negative for CD34 and HLA-DR. The CD34 and HLA-DR negativity raised the possibility of acute promyelocytic leukemia; however follow-up PML/RARA studies were all negative. Further studies revealed an AML with normal cytogenetics with FLT3-ITD and NPM1 mutations. Thereafter the patient underwent FLAG-Ida induction chemotherapy consisting of an antimetabolite (fludarabine), topoisomerase II inhibitor (idarubicin), cytarabine, and granulocyte colony-stimulating factor (G-CSF). She achieved complete remission with marrow regeneration and normalized blood cell counts and received one cycle of high-dose cytarabine consolidation. She declined allogeneic HSCT and subsequently underwent an autologous HSCT after conditioning with alkylating agents (busulfan and cyclophosphamide) which placed her in remission.

**Figure 2:**
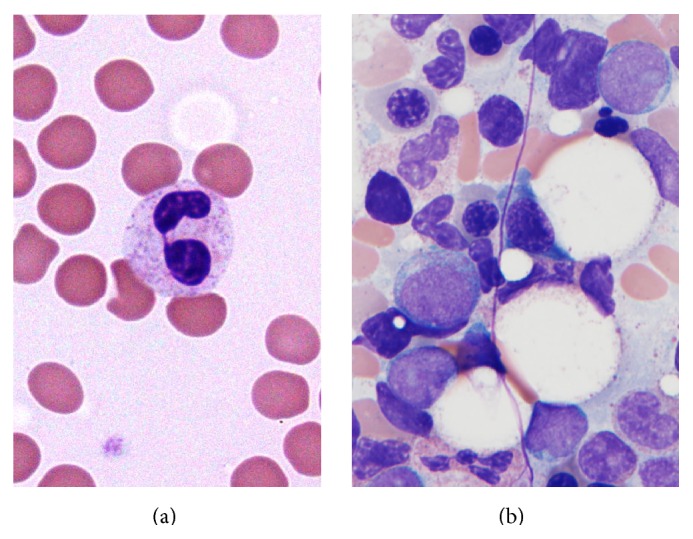
Therapy-related myelodysplastic syndrome. (a) Peripheral blood (Wright-Giemsa, 100x) showed a leukopenia with neutropenia and circulating pseudo-Pelger-Huet cells with bilobed hyposegmented nuclei. Peripheral blood also showed a mild normocytic anemia and thrombocytopenia. (b) Bone marrow aspirate (Wright-Giemsa, 100x) showing a mild increase in blasts (7% of cellularity) with a high nuclear to cytoplasmic ratio, vesicular chromatin, and prominent nucleoli with variable granularity. No Auer rods were identified. A rare dysplastic erythroid cell with a blebbed nucleus is noted.

**Figure 3:**
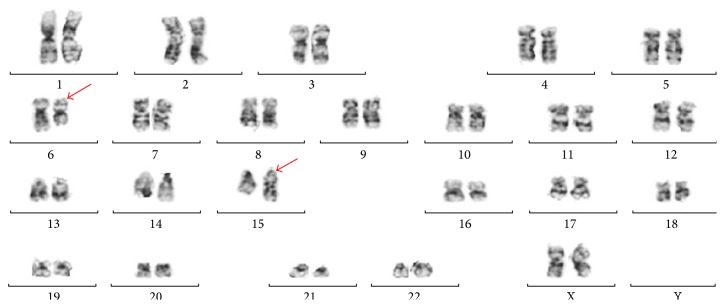
Cytogenetic karyotype. Karyotype of bone marrow aspirate cells showing t(6;15)(q12;q15). Red arrows show involved chromosomes 6 and 15.

Three years after her initial AML diagnosis and treatment, she was noted to have developed cytopenias during routine surveillance. A CBC showed a normal hemoglobin (12.9 g/dL) and leukopenia (2.6 × 10^9^/L) with neutropenia and circulating pseudo-Pelger-Huet neutrophils (Figure 2(a)) and thrombocytopenia (99 × 10^9^/L). No circulating blasts were identified. A bone marrow biopsy showed a normocellular marrow (approximately 50% cellularity) with increased blasts enumerated at 7% (Figure 2(b)) by aspirate morphology count (in the absence of G-CSF or cytokine treatment). Flow cytometry performed on the marrow showed that the blasts had a different immunophenotype than the patient's original AML, with positivity for CD34, CD117, HLA-DR, and dim CD4. No other aberrancies were noted. Trilineage hematopoiesis was present with a left shift in myeloid cells and erythroid hyperplasia. Scattered dysplastic erythroid cells with blebbed nuclei and rare dysplastic hypolobated megakaryocytes were also noted (Figure 2(b)). An iron stain on the aspirate showed occasional ring sideroblasts (<10% of the erythroid lineage). Additional studies, including T-cell and B-cell clonality assays, and FLT3-ITD and NPM1 mutation studies were all negative. However, cytogenetics revealed an abnormal single translocation, showing 46, XX, t(6;15)(q12;q15) in five of twenty-two cells (23%). The remaining seventeen cells showed a normal female complement (77%) (Figure 3). Overall, these findings support the diagnosis of therapy-related myeloid neoplasm (t-MDS), best characterized per the WHO 2008 as a refractory anemia with excess blasts-1 (RAEB-1) [1]. Following this new diagnosis, the patient received seven cycles of azacitidine. Interim bone marrow biopsies during and after azacitidine therapy showed no excess blasts. The first biopsy after four cycles of azacitidine showed resolution of the t(6;15) clone, but there was an emergence of a new trisomy 8 clone. Repeat biopsy after the seventh cycle of azacitidine showed resolution of the trisomy 8 clone and reappearance of the t(6;15) clone. The patient then underwent a matched unrelated donor allogeneic HSCT with subsequent remission and normalization of the cytogenetics and molecular findings. She remains without evidence of relapse eight months after transplant.

## 2. Materials and Methods

The Wright-Giemsa stained aspirate smears and haematoxylin and eosin (H&E) stained core bone marrow biopsies were reviewed. Immunohistochemistry studies were performed on formalin fixed, paraffin-embedded tissue with monoclonal antibodies using an automated immunostainer (Dako Omnis) using the manufacturer's instructions. Quality controls for each immunohistochemistry stain were reviewed. Flow cytometry was performed as part of routine case workup.

Standard PCR-based and cytogenetic analysis was performed at the Associated Regional and University Pathologists, Inc., ARUP Laboratories (Salt Lake City, Utah). Detection of the FLT3 TKD and IKD mutation was performed on isolated DNA using targeted fluorescent PCR primers for sequence amplification. The TKD products were cut using the EcoRV restriction enzyme and the resultant amplified ITD and TKD sequences were analyzed for base pair length on an ABI 3500xl genetic analyzer. A fragment of NPM1 exon 12 was also analyzed by targeted PCR amplification and interpreted using capillary electrophoresis. For cytogenetic studies, chromosomes were prepared from a nondiluted bone marrow aspirate collected in a heparinized syringe. The specimens were transported within 48 hours to ARUP laboratories. Each sample was cultured and suspended in metaphase. Giemsa-banded karyotyping was performed and interpreted with ISCN 2013.

## 3. Discussion

### 3.1. MDS and Therapy-Related MDS

Acquired or* de novo* events causing DNA damage produce similar disorderly conduct in cellular regulation and survival, resulting in a clonal expansion characteristic of the myelodysplastic syndromes (MDS). A subset of MDS cases with a clinical history of chemotherapy and/or radiotherapy for a preexisting neoplastic or nonneoplastic condition designates a separate classification by the WHO, denoting therapy-related myeloid neoplasms which includes therapy-related myelodysplastic syndromes (t-MDS).

The exact molecular basis of the therapy-related syndromes remains to be described, although genetic polymorphisms resulting in an increased susceptibility to DNA damage by drug metabolites and altered DNA double-stranded break repair have been proposed [2]. However, more recent studies implicate preexisting chemotherapy-resistant and age-related TP53 stem cell mutations as undergoing selective clonal expansion in the post-chemotherapeutic state. In two retrospective statistically significant studies using a cohort of 108 patients [3] and 22 patients [4], it was found that although TP53 mutations are prevalent in therapy-related neoplasms, the pattern of mutation is similar in comparison to* de novo* cases. Case reports supporting this theory have also been published [5]. These studies also found that cytotoxic exposures can cause high risk cytogenetic abnormalities in addition to other genetic and epigenetic alterations of hematopoietic stem cells that may lead to resistance, poor response to treatment, and/or relapse [3].

The patient had a past history of acute myeloid leukemia and was given drugs known to be associated with therapy-related myeloid neoplasms, such as antimetabolites, alkylating agents, and topoisomerase II inhibitors. Alkylating agents, such as cyclophosphamide and busulfan, have been shown to be associated with t-MDS in a dose-dependent manner. In a retrospective study of 306 patients by Smith et al. [6], the development of t-MDS from the first treatment dose was found to be variable within 58–73 months, multifactorially dependent on the age of the patient, chemotherapeutic agent, drug concentration, and a neoplastic versus nonneoplastic original diagnosis, with a median latency period of approximately 64 months. As per Smith et al. [6], the most common cytogenetic findings in t-MDS patients with a history of alkylating agent therapy are loss or deletion of chromosomes 5 and 7. Topoisomerase II inhibitors have been implicated in a more aggressive clinical course [7]. Topoisomerase II administration is associated with known balanced translocations including the MLL (mixed-lineage leukemia) gene at 11q23 or PML/RARA (promyelocytic leukemia/retinoic acid receptor, alpha) gene [8]. Another cause of the development of t-MDS includes a history of treatment with purine analogues such as fludarabine as used in chronic lymphocytic leukemia (CLL) patients. Plausible mechanisms include T-cell immunosuppression and ill-defined oncogenetic properties [9]. Additionally, studies have shown that elderly patients are at greater risk for t-MDS [10].

### 3.2. Therapy-Related MDS and Risk Scoring Systems

Given the history of prior exposure to anthracycline- and alkylator-based therapies three years prior to the development of MDS RAEB-1 with a novel t(6;15)(q12;q15) balanced translocation, the patient described in this report meets WHO 2008 criteria for a therapy-related myeloid neoplasm. The latency of three years and development of a clone with a balanced translocation implicate the prior anthracycline as a likely contributing agent for this patient.

A variety of prognostic scoring systems are currently utilized to risk-stratify patients with MDS to inform treatment decisions, including the International Prognostic Scoring System (IPSS), Revised IPSS (IPSS-R), World Health Organization Prognostic Scoring System (WPSS), and the Global M.D. Anderson Risk Model Score for MDS (MDAS) [11]. Patients with t-MDS are generally predicted to have a poorer prognosis than* de novo* MDS; however, the current key prognostic scoring systems were derived from patients with* de novo* MDS and excluded cases of therapy-related MDS [5]. Therefore, Ok et al. [3] studied a large cohort of t-MDS and t-AML patients and found that the IPSS-R has prognostic significance in these patient populations. Ok et al. [3] also confirmed the negative prognostic impact of the t-MDS diagnosis, as survival was inferior for each of the IPSS-R prognostic categories. Other studies have shown that t-MDS involving chromosomes 5 and/or 7 or complex karyotypes with >3 abnormalities are also associated with a poor-risk IPSS category and overall poor outcome [12].

The IPSS-R estimates overall survival and AML-free survival based on several clinical variables, including hemoglobin concentration, absolute neutrophil count, platelet count, bone marrow aspirate blast count, and bone marrow cytogenetic findings [13]. As per the IPSS-R, the reported patient was categorized as high risk due to her presentation with cytopenias, 7% bone marrow blasts, and intermediate risk cytogenetics. The high risk category is associated with a median overall survival of 1.6 years and median time to 25% transformation to AML of 1.4 years in the untreated patient cohort used to derive the IPSS-R. Based on Ok et al. [3] study, the predicted median overall survival was even less at 8.9 months. Because of the poor prognosis in this younger transplant-eligible patient, the decision was made to initiate disease-modifying therapy with allogeneic HSCT preceded by bridging azacitidine therapy. The presence of the unique t(6;15)(q12;q15) in this t-MDS and its prognostic impact remain to be found.

### 3.3. Clinical Relevance of Translocation (6;15)(q12;q15)

To our knowledge, a “sole” translocation t(6;15)(q12;q15) in a patient with t-MDS has not been reported in the literature. The rare reported cases of t(6;15) are noted only as secondary cytogenetic findings in association with certain myeloid neoplasms and other nonhematopoietic tumors. Chromosomal translocations involving 6 and 15 have been reported in clinically recognizable congenital anomalies [14] and solid organ tumors such as Wilms tumor, mesothelioma, and adenocarcinoma [15].

Specifically, only a few reports are available for hematopoietic associated t(6;15) neoplasms but these are not sole translocations, rather secondary cytogenetic findings which include Ph-chromosome positive cases of chronic myelogenous leukemia [16] and acute promyelocytic leukemia [17]. Current literature lacks detailed documentation of chromosomes 6q12 and 15q15 translocations in myeloid malignancies. Candidate genes on 6q12 that may be implicated in t(6;15)(q12;q15) translocations include the gene encoding stromal cells influencing erythropoietic development SMAP1, the zinc finger PHF3 in association with corepressor proteins of the JARID demethylase family, and the PTP4A1 gene encoding a nuclear tyrosine phosphatase involved in cellular proliferation. Additionally, the gene TYRO3 located on 15q15 is part of the receptor tyrosine kinase TAM gene subfamily [18] which may also play a role in this particular translocation. Thus, although the mechanisms of disease progression in t(6;15) remain unknown, they may be related to the above genes or the function of related tumor suppressor genes that have yet to be identified on chromosome 6q12 or 15q15. It is possible that this balanced translocation could have led to a tumor suppressor gene deletion presumably precipitating the patient's disease [19].

## 4. Conclusion

With the advancement of cancer treatments and therapeutic regimens, the incidence of t-MDS has increased. Although current prognostic scoring systems have been validated in t-MDS, there is a need for newer classification schema that better distinguishes the prognosis of t-MDS compared to* de novo* MDS [3]. Importantly, current studies are evaluating molecular mutations to risk-stratify these patients. Herein, we report the first case of MDS or t-MDS with t(6;15)(q12;q15) which to our knowledge has not been described before. Although the prognostic significance of t(6;15) in t-MDS remains unclear, this patient's favorable clinical course and response to treatment may be of value for future studies which can be correlated as we enhance our understanding of these disease processes.
